# Ferroptosis-related genes LPCAT3 and PGD are potential diagnostic biomarkers for osteoarthritis

**DOI:** 10.1186/s13018-023-04128-2

**Published:** 2023-09-19

**Authors:** Lufei Wang, Shouxiu Ye, Jianliang Qin, Min Tang, Ming-You Dong, Jie Fang

**Affiliations:** 1 Guangxi Key Laboratory of Oral and Maxillofacial Rehabilitation and Reconstruction & Department of Orthodontics, College and Hospital of Stomatology, Guangxi Medical University, Nanning, China; 2 The Key Laboratory of Molecular Pathology (For Hepatobiliary Diseases) of Guangxi, Affiliated Hospital of Youjiang Medical University for Nationalities, Baise, China; 3 State Key Laboratory of Oral Diseases and National Clinical Research Center for Oral Diseases and Department of Orthodontics, West China Hospital of Stomatology, Sichuan University, Chengdu, China

**Keywords:** Osteoarthritis, Ferroptosis, Bioinformatic analysis, Diagnostic biomarker, Immune infiltration

## Abstract

**Background:**

Osteoarthritis (OA) is the most common chronic joint disease and how ferroptosis contributes to OA has garnered much attention recently. Bioinformatics promoted the discovery of ferroptosis-related biomarkers for OA. But since OA is a whole-joint disease, sensitive biomarkers for OA are still limited. We herein focused on subchondral bone, a joint component often-ignored by existing bioinformatic reports, to identify ferroptosis-related diagnostic biomarkers for OA.

**Method:**

Microarray datasets GSE51588 and GSE55457 were downloaded from Gene Expression Omnibus database. Ferroptosis-related differential expression genes (Ferr-DEGs) between OA and normal samples were identified and their functional enrichment was analyzed. Common genes for OA diagnosis were selected from Ferr-DEGs using the combination of SVM-RFE, LASSO regression, and RandomForest machine learning algorithms. Common genes' diagnostic value was verified by receiver operating characteristic (ROC) curve and their association with immune infiltration was analyzed by CIBERSORT. Finally, candidate gene’s expression was verified in chondrocytes from OA patients and in an in vitro IL-1β-induced OA model, by RT-PCR.

**Results:**

Two ferroptosis-related genes, *LPCAT3* and *PGD*, were identified as OA diagnostic biomarkers and confirmed by ROC diagnostic test. The association of *LPCAT3* and *PGD* with the infiltration of several types of immune cells was identified. The decreased expression of *LPCAT3* and *PGD* was both confirmed in OA chondrocytes and IL-1β-induced OA condition.

**Conclusions:**

We identified ferroptosis-related genes *LPCAT3* and *PGD* as potential diagnostic biomarkers for OA, which may offer insight into the role of ferroptosis in OA and provides useful information for the diagnosis and treatment of OA.

## Background

Osteoarthritis (OA) is the most common chronic joint disease characterized by cartilage damage, subchondral bone destruction, and synovium inflammation [1]. It is estimated that more than 240 million people worldwide suffer from OA, and OA prevalence is even rising due to population aging, which causes a financial burden for patients and society [2]. Several OA-related proteins, such as type 2 collagen (COL2A1), aggrecan (ACAN), and matrix metalloproteinase (MMP)-13, have been commonly acknowledged as OA biomarkers [3]. However, the pathogenesis of OA is very complex in which mechanical loading, inflammation, and metabolic factors play a critical role corporately. Thus, the discovery of sensitive biomarkers, especially through high-throughput analysis, not only benefits the diagnosis and therapy but also helps understand the pathogenic mechanism of OA [4].

Ferroptosis, first discovered in 2012, is a new type of programmed cell death that is distinct from apoptosis, pyroptosis, and necroptosis in terms of morphological and biochemical features [5]. Ferroptosis involves metabolisms of lipid, iron, and thiol, and is caused by the iron-dependent accumulation of lipid peroxidation that reaches the cellular lethal level. The number of studies about the role played by ferroptosis in OA started growing rapidly from two years ago, making it a research hotspot nowadays [6, 7]. Emerging evidence has suggested that iron dyshomeostasis and lipid peroxidation contribute to OA pathogenesis and ferroptosis could be a promising target in OA therapy.

In modern medicine, high-throughput sequencing combined with bioinformatic analysis becomes a popular research tool due to its strong power in uncovering valuable clues for the diagnosis, prognosis, and treatment of diseases. Increasing interest has been aroused to reveal diagnostic markers or therapeutic targets for OA based on bioinformatic data mining. For example, in the synovium or cartilage of the osteoarthritic joint, several ferroptosis-related genes such as SLC3A2, ATF3, IL6, etc. have been suggested as OA biomarkers in this way [8, 9].

However, sensitive biomarkers for OA are still limited and OA is a whole-joint disease involving cartilage, synovium, subchondral bone, and infrapatellar fat pad. Additionally, integrative analysis using multi-algorithms plus post hoc experimental validation is often necessary for obtaining more reliable predictions. Thus, focusing on subchondral bone, an easily-ignored joint component, we combined three machine learning algorithms to identify ferroptosis-related diagnostic biomarkers for OA and validated them in vitro, which may offer insight into the function of ferroptosis in OA together with the diagnosis and treatment of OA.

## Methods

### Data acquisition and processing

GSE51588 and GSE55457 microarray datasets were downloaded from Gene Expression Omnibus (GEO) data repository (https://www.ncbi.nlm.nih.gov/geo/). GSE51588 dataset contains 40 OA samples and 10 normal samples of human tibial plateaus (subchondral bone); GSE55457 dataset contains 10 OA samples and 10 normal samples of human synovium. The data was processed by mapping the probes to the gene names and log2-transforming quantile-normalized signal strength. Null probes were removed; replicate probes were mapped to the same gene.

### Identification of ferroptosis-related differential expression genes (Ferr-DEGs)

Gene expression differences between OA and normal samples in GSE51588 dataset were analyzed by using R package “limma” (adjusted *P* value < 0.05). The entire set of differential expression genes (DEGs) was then intersected with the ferroptosis-related gene list retrieved from FerrDb database [10] (http://www.zhounan.org/ferrdb/) that contains 621 defined ferroptosis gene regulators, to obtain the Ferr-DEGs.

### Functional enrichment analysis of the Ferr-DEGs

Gene Ontology (GO) and Kyoto Encyclopedia of Genes and Genomes (KEGG) enrichment analyses were performed to explore the major biological attributes of Ferr-DEGs. Enrichment maps were visualized by R packages “ggplot2” and “GOplot”. Enrichment analysis for Disease Ontology (DO) terms was run using R packages “clusterProfiler” and “DOSE”.

### Selection of common genes for OA diagnosis

Potential OA diagnostic biomarkers were selected from the above Ferr-DEGs, via using the combination of three machine learning algorithms: support vector machine recursive feature elimination (SVM-RFE), the least absolute shrinkage and selection operator (LASSO) regression, and RandomForest. SVM-RFE is a machine learning technique that trains a subset of features from different categories to shrink the feature set and then backward select the most predictive features [11]. LASSO regression was performed using R package “glmnet” to select the valuable variables in the linear model. The response type was set to binomial and “one standard error” rule was applied. RandomForest algorithm was applied to rank the candidate genes [12]: relative importance value > 0.25 was classified as a typical chance cause. Finally, the most predictive genes were determined as the intersection of the results from SVM-RFE, LASSO regression, and RandomForest.

### Diagnostic value of the candidate genes in OA

The comparison of candidate gene’s expression between OA and control group was determined by Student’s t test. Diagnostic value of the candidate genes was analyzed by receiver operating characteristic (ROC) curve in GSE51588 dataset.

For external validation, we screened all of the existing GEO datasets that contains osteoarthritic subchondral bone samples but did not find a suitable dataset for our study. Thus, we selected GSE55457 dataset, which contains 10 OA samples and 10 normal samples of human synovium, for external validation.

### Immune cell infiltration analysis

To evaluate the role of immune microenvironment in OA development, CIBERSORT algorithm [13] (http://cibersortx.stanford.edu), which can perform linear support vector regression to deconvolute gene expression profiles, was employed to estimate the abundance of 22 infiltrated immune cell types in each sample. Student’s t test was employed to compare the difference in immune cell infiltration between the OA and the normal groups. Then, the correlation of the candidate genes to the abundance of infiltrated immune cells was determined by Spearman’s rank correlation test and visualized by R package “ggplot2”.

### Experimental validation of the candidate genes

Chondrocytes from healthy people (CP-H107, Procell Life Science & Technology, Wuhan, China) and OA patients (402OA-05A, Cell Applications) were used for experimental validation of the candidate genes. Cells were maintained in DMEM/F12 medium (HyClone) supplemented with 10% fetal bovine serum at 37 °C, 5% CO_2_. Passage 2–3 was used for experiments and then cells were harvested for RT-PCR.

Additionally, we also performed an inflammatory-cytokine-induced OA model. Interleukin-1β (IL-1β) is one of the most popular cytokines for modeling OA in vitro because it can induce cartilage destruction through promoting the release of matrix metalloproteinases from chondrocytes [14]. We used various concentrations of IL-1β (1, 5, 20 ng/ml where indicated) to stimulate CP-H107 chondrocytes and harvested the cells for RT-PCR after 48 h.

### RT-PCR detection

The expression of candidate genes and OA marker genes (*COL2A1*, *ACAN*, *MMP13*) was detected using RT-PCR. Cells were lysed by TRIzol (Invitrogen) for total RNA extraction. cDNA reverse-transcription was performed by using iScript™ cDNA Synthesis Kit (Bio-Rad). RT-PCR was carried out on StepOnePlus RT-PCR system (Applied Biosystems) using the iTaq™ Universal SYBR Green Supermix reagent (Bio-Rad). The relative expression of target genes was normalized to glyceraldehyde-3-phosphate dehydrogenase (GAPDH) based on the ΔΔCT method. Primers are listed in Table 1.

**Table 1:** Primer sequences for RT-PCR

Gene	Forward sequence (5′–3′)	Reverse sequence (5′–3′)
*LPCAT3*	GGAGCTGAGCCTTAACAAGTT	CAAAGCAAAGGGGTAACCCAG
*PGD*	ATGGCCCAAGCTGACATCG	AAAGCCGTGGTCATTCATGTT
*COL2A1*	TGGACGATCAGGCGAAACC	GCTGCGGATGCTCTCAATCT
*ACAN*	CCCCTGCTATTTCATCGACCC	GACACACGGCTCCACTTGAT
*MMP13*	CCAGACTTCACGATGGCATTG	GGCATCTCCTCCATAATTTGGC
*GAPDH*	GGAGCGAGATCCCTCCAAAAT	GGCTGTTGTCATACTTCTCATGG

### Statistical analysis

R program 4.3.1 was used for bioinformatic analysis and statistical computing. The comparisons between two groups were determined by Student’s *t* test while multiple-group comparisons were determined by one-way ANOVA plus Bonferroni post hoc test. Correlations were determined by Spearman’s rank correlation test. *P* < 0.05 was considered significant. For experimental validation assays, experiments were repeated three times and data were presented as mean ± SEM.

## Results

### Identification of Ferr-DEGs and their functional enrichment

**Fig. 1:**
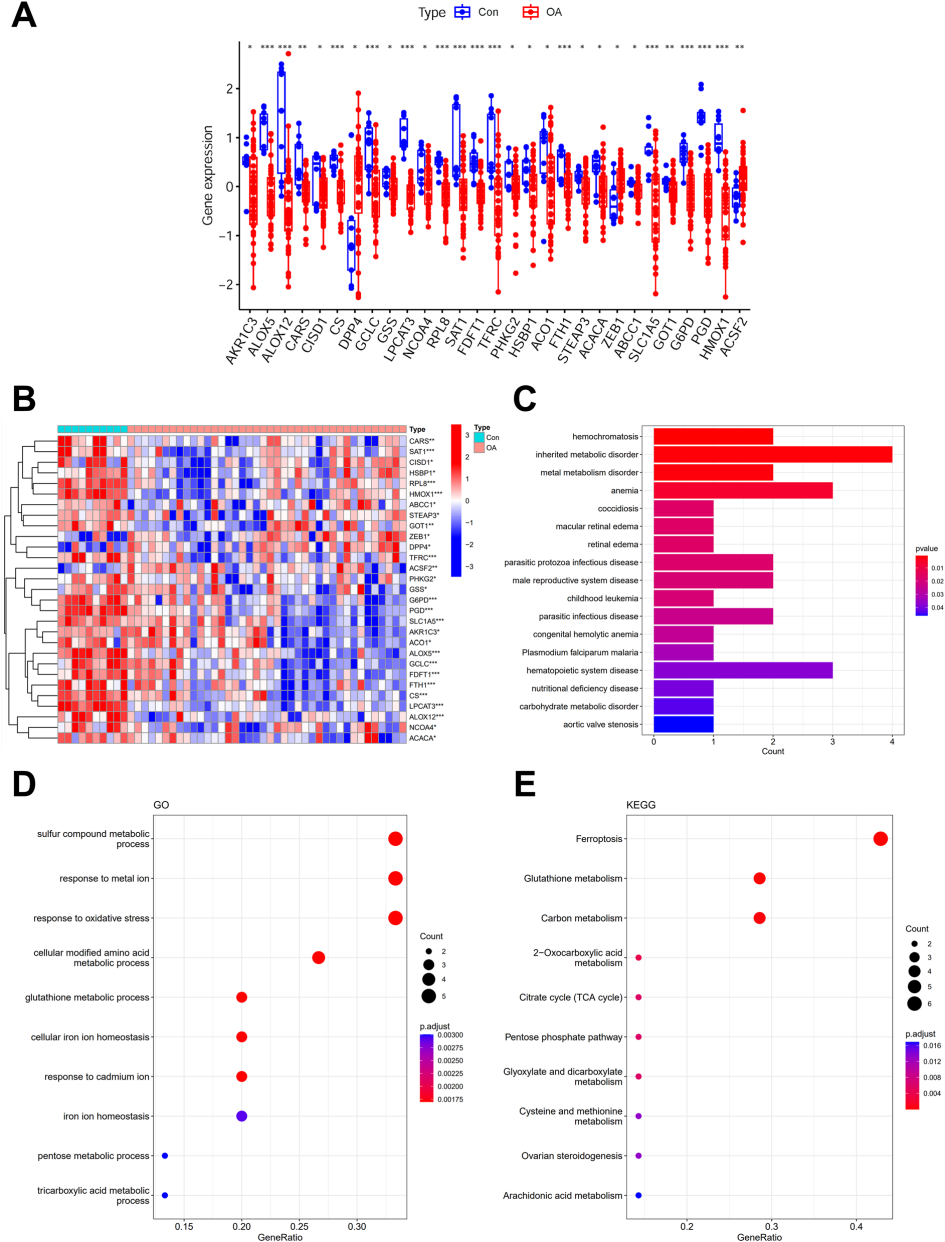
Ferr-DEGs and their functional enrichment. **A** Gene expression level of 29 Ferr-DEGs identified from GSE51588 microarray dataset. *indicates significance. **B** Ferr-DEGs expression heatmap in each sample. *indicates significance when OA vs. normal control. **C**–**E** DO, GO, and KEGG top 10 enriched pathways for Ferr-DEGs

We used GSE51588 microarray dataset, which contains the transcriptome profile of human knee tibial plateaus (40 OA and 10 normal samples), to identify Ferr-DEGs. Ferr-DEGs were defined as the intersection of entire DEGs (adjusted P value < 0.05) and FerrDb-defined ferroptosis-related gene list [10]. In total, there were 29 Ferr-DEGs in the OA group compared to the normal control group, including 3 upregulated and 26 downregulated genes (Fig. 1A, 1B).

In an attempt to investigate the biological activities and pathways associated with Ferr-DEGs among the OA cases, DO, GO, and KEGG enrichment analyses were performed. The top 10 enriched pathways for DO, GO, and KEGG terms, respectively, can be seen in Fig. 1C-1E. Taken DO, GO, and KEGG enrichment results together, metabolism-related pathways presented most frequently. For example, the “inherited metabolic disorder”, “metal metabolism disorder” and “carbohydrate metabolic disorder” in DO enrichment result (Fig. 1C); the “sulfur compound metabolic process”, “cellular modified amino acid metabolic process”, etc. in GO enrichment result (Fig. 1D); the “glutathione metabolism”, “carbon metabolism” etc. in KEGG enrichment result (Fig. 1E).

### Selection of ferroptosis-related diagnostic biomarkers for OA

**Fig. 2:**
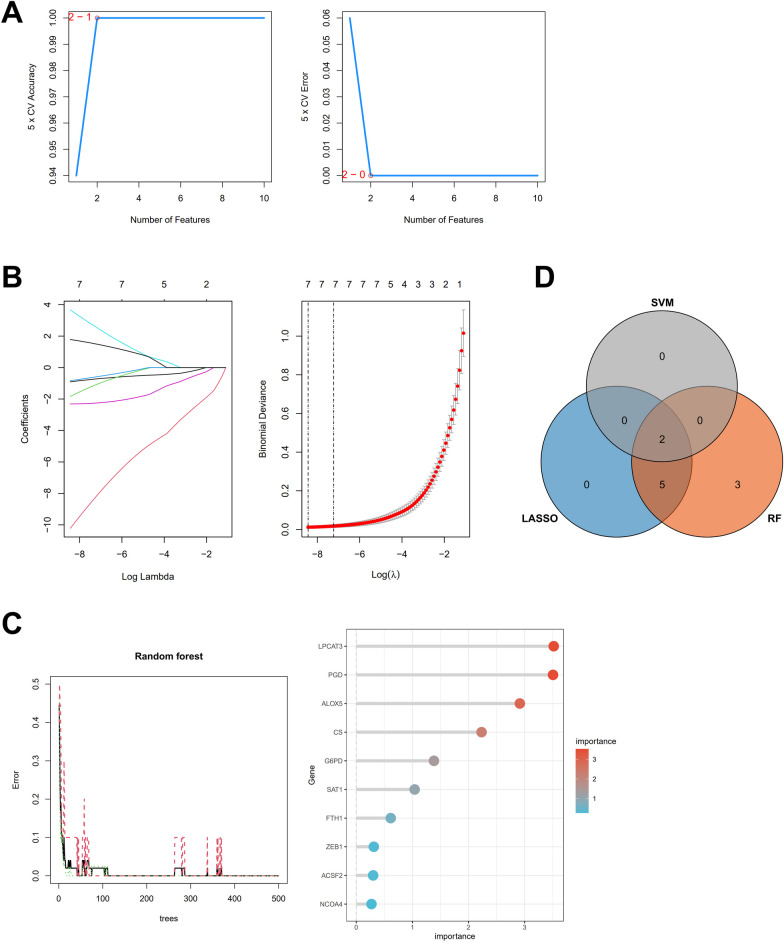
Selection of candidate genes from Ferr-DEGs for OA diagnosis. **A** Candidate gene expression validation by SVM-RFE selection. **B** Adjustment of feature selection in LASSO regression model. **C** RandomForest: error rate versus the number of classification trees; list of relatively important genes. **D** Venn diagram shows 2 common genes shared by SVM-RFE, LASSO, and RandomForest methods

Aiming to obtain more reliable candidates, we used three machine learning algorithms to determine OA diagnostic biomarkers from Ferr-DEGs. SVM-RFE selected the most 2 predictive features (Fig. 2A) while LASSO regression selected 7 predictive genes from statistically significant univariate variables (Fig. 2B). RandomForest algorithm calculated the relationship between the error rate and the number of classification trees and ranked 10 genes with relative importance > 0.25 (Fig. 2C). We used a Venn diagram to display the intersection of three methods and eventually found 2 common genes, lysophosphatidylcholine acyltransferase 3 (LPCAT3) and phosphogluconate dehydrogenase (PGD), as potential OA diagnostic biomarkers (Fig. 2D).

### Common gene’s diagnostic value for OA

**Fig. 3:**
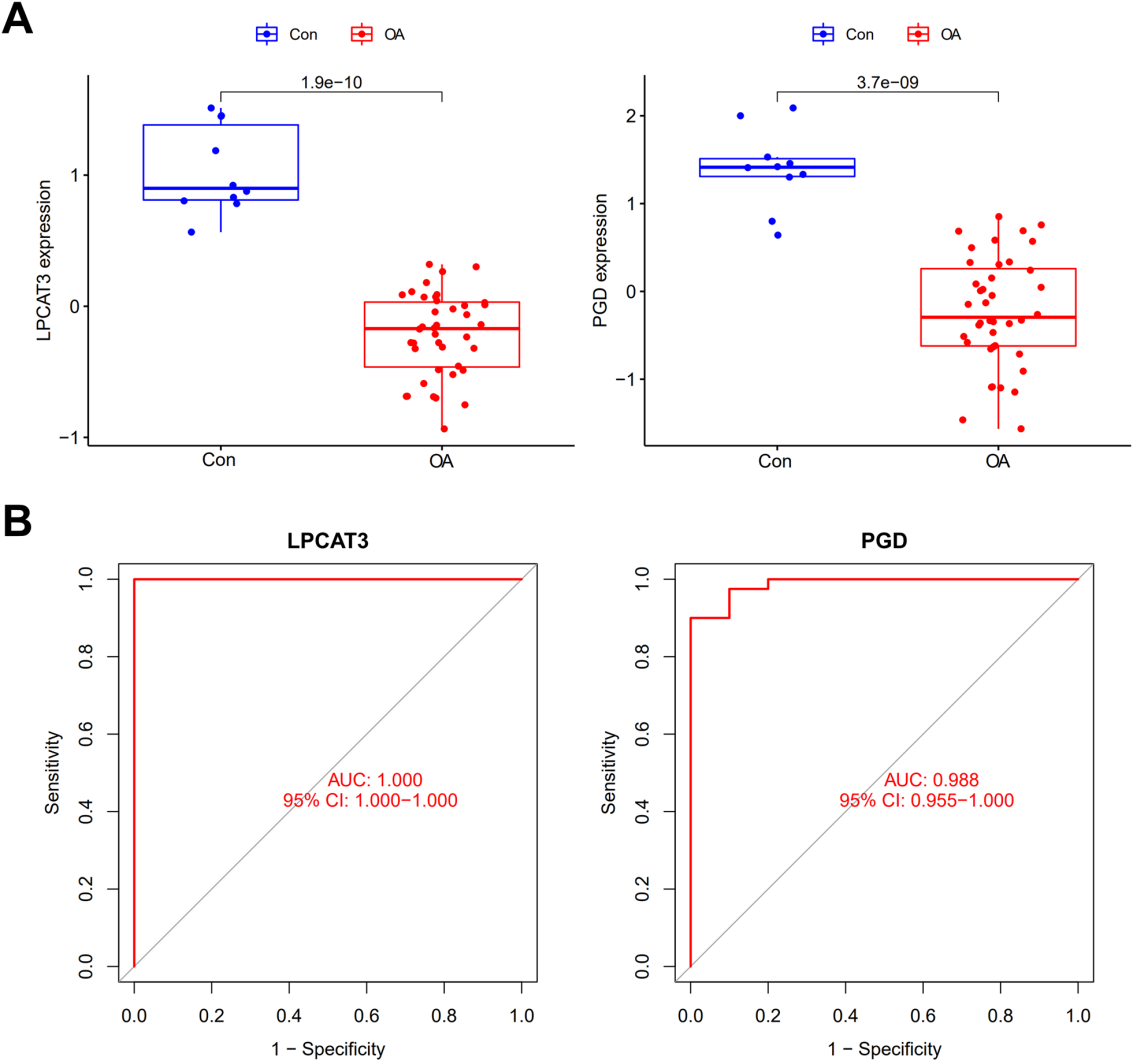
Expression pattern and diagnostic value of the candidate genes in training set GSE51588. **A** Expression of *LPCAT3* and *PGD* in OA compared to normal control. **B** ROC curve for OA diagnosis

We next verified the diagnostic value of these two common genes *LPCAT3* and *PGD* for OA. In training set GSE51588 (Fig. 3), decreased expression of *LPCAT3* and *PGD* was observed in OA condition and both of them showed a very good diagnostic accuracy for OA.

**Fig. 4:**
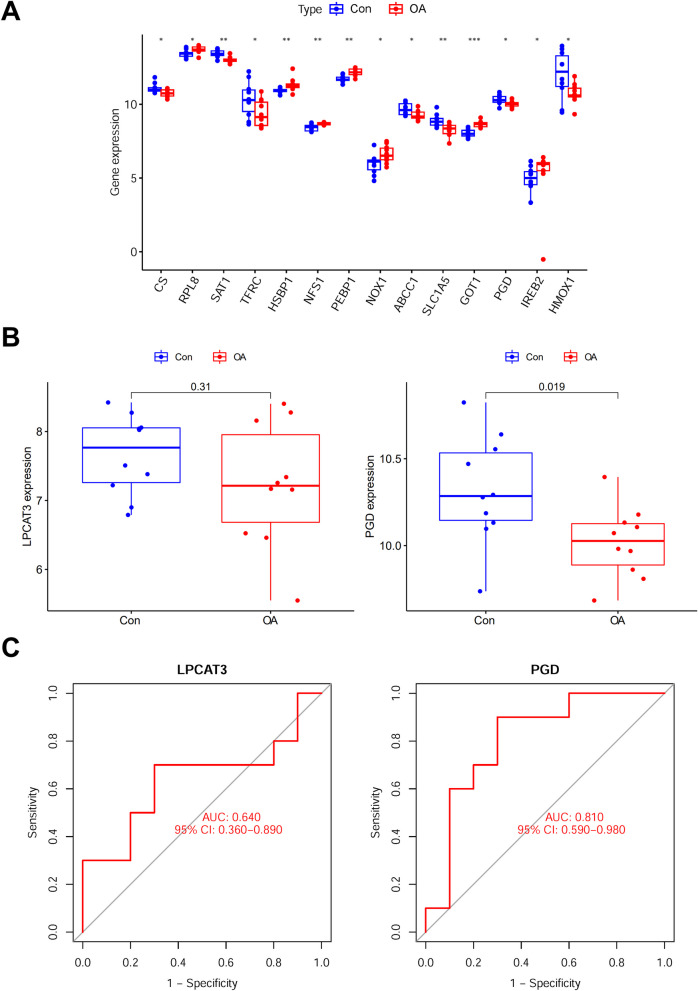
Expression pattern and diagnostic value of the candidate genes in external validation set GSE55457. **A** Gene expression level of Ferr-DEGs. *indicates significance. **B** Expression of *LPCAT3* and *PGD* in OA compared to normal control. (C) ROC curve for OA diagnosis

We also verified the diagnostic value of *LPCAT3* and *PGD* in an external validation set GSE55457 that contains human synovium samples because no suitable subchondral bone GEO dataset was found. 14 Ferr-DEGs were found: 6 upregulated and 8 downregulated (Fig. 4A). In validation set GSE55457, *LPCAT3* showed a decrease trend in OA, though not significant, and *PGD* expression was significantly decreased in OA (Fig. 4B), which is generally consistent with that of GSE51588. While *LPCAT3* showed a poor diagnostic accuracy, *PGD* still showed a good diagnostic accuracy (Fig. 4C).

### Immune cell infiltration pattern in OA and its association with the candidate genes

**Fig. 5:**
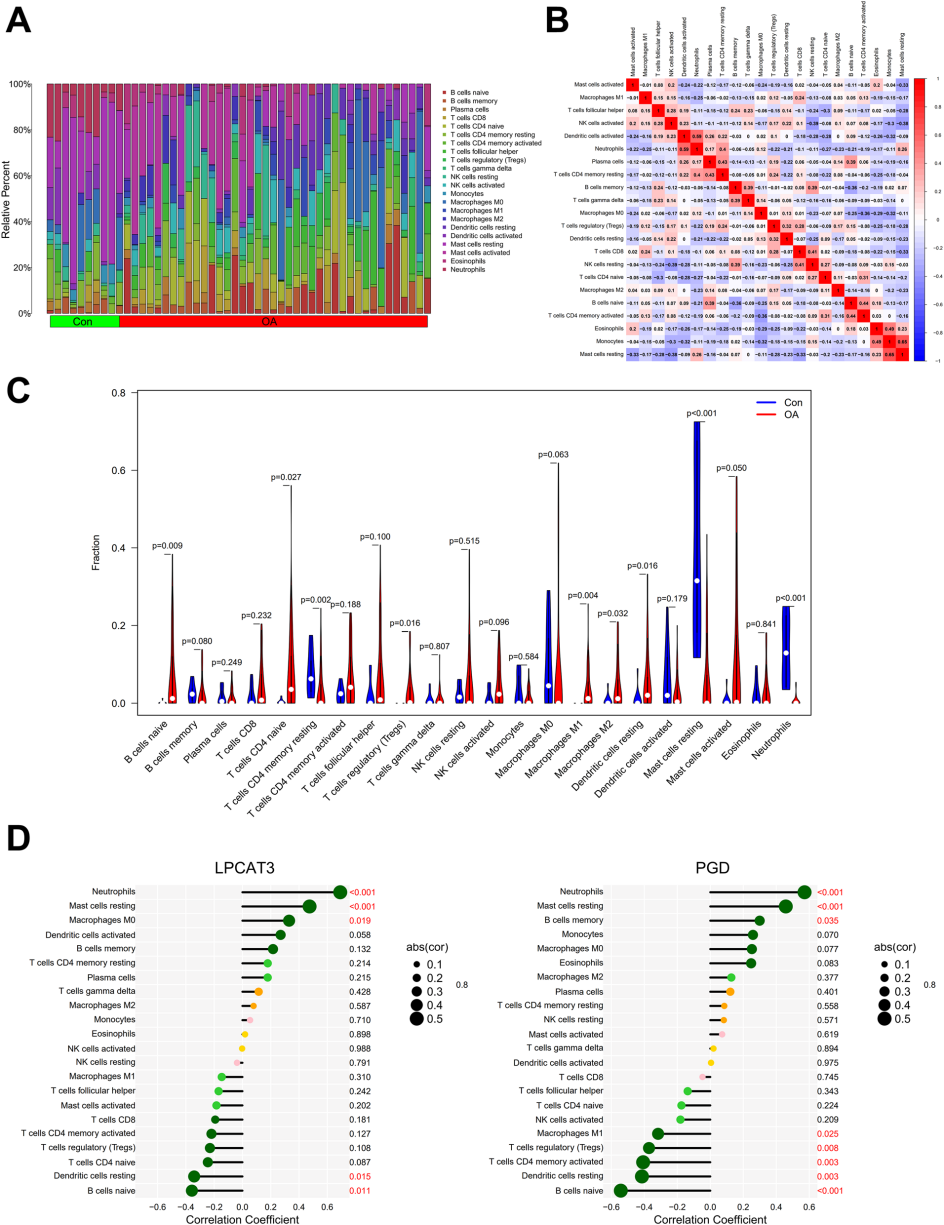
Immune cell infiltration in OA. **A** Relative abundance of 22 types of infiltrated immune cells in OA and normal control samples. **B** Correlation matrix of immune cells. **C** Immune cell infiltration comparison between OA and normal control. **D** Correlation of *LPCAT3* or *PGD* to infiltrated immune cells in OA

Inflammation and immune microenvironment play a critical role in OA pathogenesis. CIBERSORT-based immune cell infiltration analysis is a powerful tool to provide references for the prediction of disease course [13]. We used CIBERSORT to generate the landscape of immune cell infiltration in the OA and the normal control samples in GSE51588 dataset, which displays the relative proportion of 22 types of immune cells (Fig. 5A). Correlations between every two cell types can be seen in Fig. 5B correlation matrix. As Fig. 5C shows, compared to the normal control, the OA group had higher infiltration of B cells naïve, T cells CD4 naïve, T cells regulatory, macrophages M1, macrophages M2, and dendritic cells resting. By contrast, the OA group had lower infiltration of T cells CD4 memory resting, mast cells resting, and neutrophils.

We next analyzed the association of *LPCAT3* or *PGD* with immune cell infiltration in OA (Fig. 5D). The infiltration of several types of immune cells was found to have a significant correlation to *LPCAT3* or *PGD* expression. Among them, both *LPCAT3* and *PGD* had a positive correlation to neutrophils and mast cells resting (*P* < 0.05), while having a negative correlation to B cells naïve and dendritic cells resting (*P* < 0.05). This implies that *LPCAT3* and *PGD* might have an important function on these immune cells and thus contribute to the pathogenesis of OA, which can be a good source for future studies.

### In vitro* validation of LPCAT3 and PGD as OA diagnostic biomarkers*

**Fig. 6:**
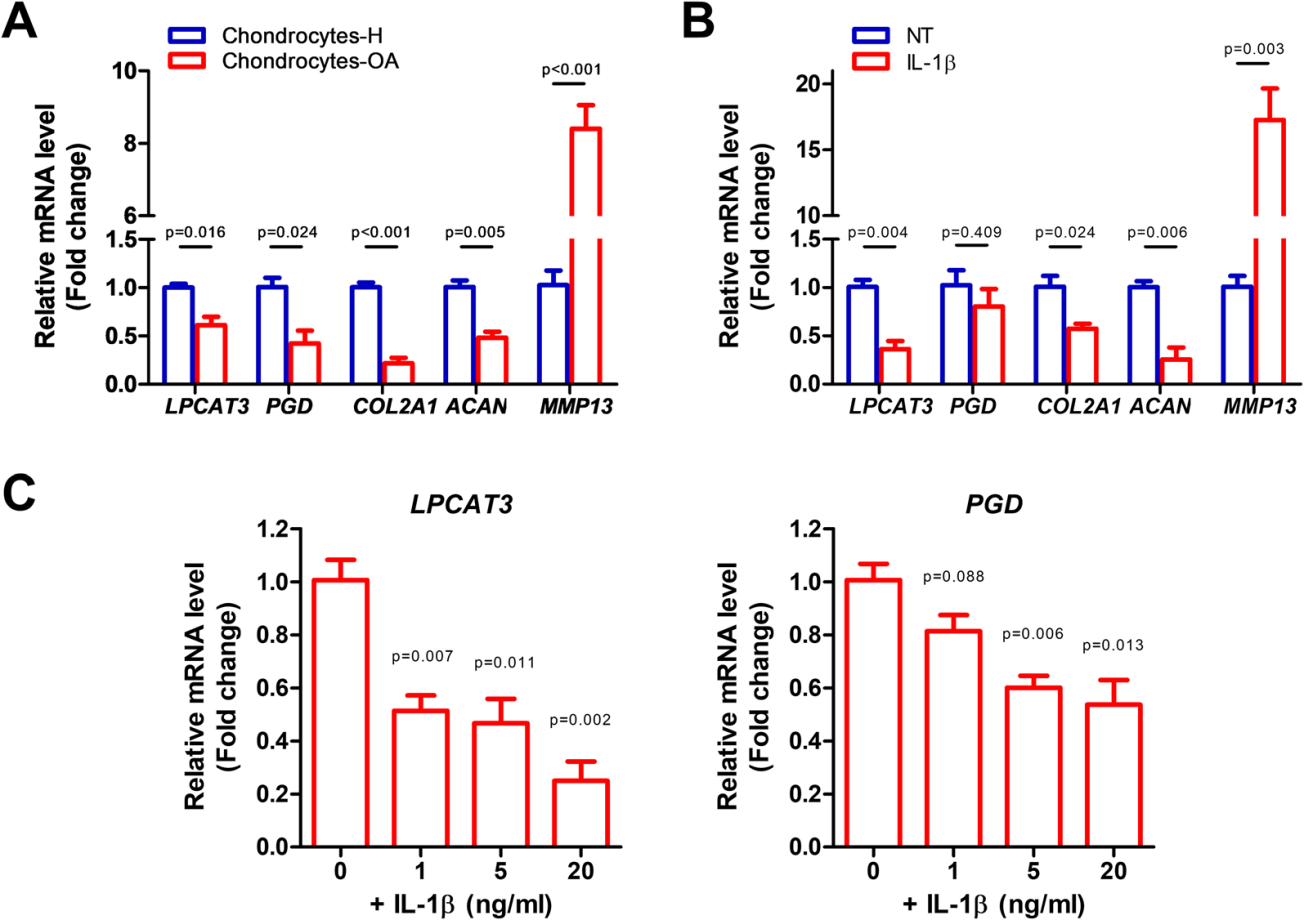
In vitro validation of *LPCAT3* and *PGD* as OA diagnostic biomarkers. **A** RT-PCR detection of *LPCAT3* and *PGD* in chondrocytes-H (CP-H107 chondrocytes from healthy people) and chondrocytes-OA (402OA-05A chondrocytes from OA patients). **B** RT-PCR detection of *LPCAT3* and *PGD* in IL-1β-induced OA model. 1 ng/ml IL-1β stimulated CP-H107 chondrocytes for 48 h. **C** Responses of *LPCAT3* and *PGD* expression to IL-1β concentrations, as detected by RT-PCR. Various concentrations of IL-1β stimulated CP-H107 chondrocytes for 48 h. *n* = 3 for all experiments; internal control GAPDH.

We next validated *LPCAT3* and *PGD* as OA diagnostic biomarkers in in vitro OA models. First, we detected *LPCAT3* and *PGD* expression in chondrocytes from healthy people and OA patients (Fig. 6A). Chondrocytes-OA (402OA-05A chondrocytes from OA patients) are a useful OA model and showed a robust OA-like phenotype, demonstrated by the decreased expression of *COL2A1* and *ACAN* and the increased expression of *MMP13*. Both *LPCAT3* and *PGD* expression were reduced in chondrocytes-OA, which confirmed our bioinformatic findings.

We further confirmed *LPCAT3* and *PGD* expression in an IL-1β-induced OA model. IL-1β stimulation on chondrocytes is one of the most popular models for studying OA in vitro [14]. The OA-like response in CP-H107 chondrocytes upon IL-1β treatment (decreased expression of *COL2A1* and *ACAN* and increased expression of *MMP13*) demonstrated the reliability of our experimental system (Fig. 6B). In IL-1β-induced OA condition, decreased *LPCAT3* expression was confirmed. *PGD* expression also showed a decrease trend, though not statistically significant. Notably, the expression of *LPCAT3* and *PGD* decreased as IL-1β treatment concentration increased (Fig. 6C), suggesting a negative association between *LPCAT3* and *PGD* expression and the severity of OA.

Collectively, although *LPCAT3* did not show good diagnostic accuracy in external validation set (Fig. 4), these in vitro results strongly supported the diagnostic value of *LPCAT3* and *PGD* so we propose *LPCAT3* and *PGD* as potential biomarkers for OA.

## Discussion

Over the past two years, studies focused on the role of ferroptosis in OA were increasing rapidly and thus made this a research hotspot. Clinical observations found that iron accumulation was commonly detected in the synovial fluid, synovia, and cartilage of OA patients and was positively correlated with OA severity [15, 16]. Ferroptotic alternations, such as iron accumulation, lipid peroxidation, and mitochondrial dysfunction are closely associated with OA progression where GPX4-mediated oxidative stress and extracellular matrix degradation serve as a core mechanism [16–18]. Nevertheless, how ferroptosis contributes to OA pathogenesis remains to be fully elucidated. In recent years, bioinformatics data mining greatly accelerated the discovery of new molecular targets for OA therapy. Xia et al. applied protein–protein interaction (PPI) network algorithm on a synovial tissue gene array dataset and screened 7 ferroptosis-related genes (*ATF3*, *IL6*, *CDKN1A*, *IL1B*, *EGR1*, *JUN*, *CD44*) to be possible biomarkers and therapeutic targets for OA [8]. Liu et al. identified ferroptosis-related gene *SLC3A2* as a therapeutic target for OA, via performing weighted correlation network analysis (WGCNA) and PPI analysis on a cartilage tissue RNA-seq dataset [9]. Considering OA is a whole-joint disease involving cartilage, synovium, subchondral bone, and infrapatellar fat pad, our findings based on a subchondral bone tissue gene array is a good supplement to the previous synovium- and cartilage-derived discoveries [8, 9]. Additionally, in order to obtain more reliable outcomes, we combined the results from 3 machine learning algorithms (SVM-RFE, LASSO, RandomForest) and validated the common genes in vitro.

LPCAT3 is an enzyme that involves in the synthesis and decomposition of polyunsaturated fatty acids and can mediate the sensitivity of cells to ferroptosis [19]. To date, we did not find any reports about its association with arthritis. By contrast, PGD is a vital oxidative carboxylase in the pentose phosphate pathway and its role in arthritis only has very limited reports. For example, salivary PGD level was altered in patients with rheumatoid arthritis compared to healthy subjects [20]. PGD activity in synovial lining cells was suppressed by menadione epoxide administration in an experimental immune arthritis model [21]. Based on the above, the role of LPCAT3 and PGD remains largely unknown in OA so it is worthy of further investigations.

Immune microenvironment is closely associated with OA development and immune cell infiltration in joint tissues can be significantly altered under OA condition [8, 22]. We found that the immune cell infiltration pattern in osteoarthritic subchondral bone was partly consistent with the results in osteoarthritic synovium or cartilage reported by other studies [8, 22]. We also revealed that ferroptosis-related genes *LPCAT3* and *PGD* were correlated to the infiltration of several types of immune cells, especially neutrophils, mast cells resting, B cells naïve, and dendritic cells resting. This implies that *LPCAT3* and *PGD* may contribute to OA pathogenesis via regulating the activities of these cells, which is a good point to test in the future.

Of course, this study also has some limitations. First, only one GEO dataset was included for bioinformatic analysis and the sample size is not quite large, which sometimes might cause bias. Future studies with a much larger sample size or if can combine multiple datasets are helpful to improve the validity. Second, comprehensive validation experiments, especially in vivo and clinical investigations, can greatly elevate the diagnostic and therapeutic values of the OA biomarkers identified herein and thus are worth exploring in the future.

In conclusion, based on integrative bioinformatic analysis and in vitro experimental validation, we identified ferroptosis-related genes *LPCAT3* and *PGD* as potential diagnostic biomarkers for OA and revealed their association with immune cell infiltration in OA. These findings may offer insight into the role of ferroptosis in OA pathogenesis and also provide useful information for the diagnosis and treatment of OA.

## Data Availability

The datasets presented in this study can be found in online repositories (GEO data repository, https://www.ncbi.nlm.nih.gov/geo/, accession number GSE51588 and GSE55457). Further inquiries can be directed to the corresponding author.

## Abbreviations

OA: Osteoarthritis
COL2A1: Type 2 collagen
ACAN: Aggrecan
MMP: Matrix metalloproteinase
GEO: Gene Expression Omnibus
Ferr-DEGs: Ferroptosis-related differential expression genes
DEGs: Differential expression genes
GO: Gene ontology
KEGG: Kyoto Encyclopedia of Genes and Genomes
DO: Disease Ontology
SVM-RFE: Support vector machine recursive feature elimination
LASSO: The least absolute shrinkage and selection operator
ROC: Receiver operating characteristic
IL-1β: Interleukin-1β
GAPDH: Glyceraldehyde-3-phosphate dehydrogenase
LPCAT3: Lysophosphatidylcholine acyltransferase 3
PGD: Phosphogluconate dehydrogenase
